# Partial replacement of red and processed meat with legumes: a modelling study of the impact on nutrient intakes and nutrient adequacy on the population level

**DOI:** 10.1017/S1368980022002440

**Published:** 2022-11-07

**Authors:** Niina E Kaartinen, Heli Tapanainen, Mirkka Maukonen, Essi Päivärinta, Liisa M Valsta, Suvi T Itkonen, Anne-Maria Pajari, Satu Männistö

**Affiliations:** 1 Department of Public Health and Welfare, Finnish Institute for Health and Welfare (THL) Mannerheimintie 166, P.O. Box 30, FI-00271 Helsinki, Finland; 2 Department of Food and Nutrition, University of Helsinki, Helsinki, Finland

**Keywords:** Legumes, Nutrient adequacy, Red meat, Sustainability, Usual intake modelling

## Abstract

**Objective:**

The shift towards plant-based diets with less meat and more legumes is a global target and requires an understanding of the consequences of dietary adequacy on the population level. Our aim was to model the impact of partial replacement of red and processed meat with legumes on nutrient intakes and population shares below dietary reference intakes.

**Design:**

Modelling study with three scenarios anchored in meat cut-offs: ≤ 70 g/d (Finnish dietary guideline); ≤ 50 g/d (Danish dietary guideline); ≤ 30 g/d (EAT-Lancet recommendation). In all subjects, the amount of meat in grams over the cut-off was replaced with the same amount of legumes. The SPADE method was used to model usual intake distributions. Meaningful differences in average intakes and in population shares below dietary reference intakes compared to the reference (FinDiet) were evaluated based on non-overlapping 95 % CI.

**Setting:**

Finnish national food consumption survey (FinDiet 2017).

**Subjects:**

Finnish adults (*n* 1655) aged18–74 years (47 % men).

**Results:**

The scenarios introduced increases in the average intakes of fibre, folate, K, Mg, Cu and Fe, and decreases in intakes of saturated fat, niacin, vitamin B_12_, Se and Zn. Meaningful shifts of the usual intake distributions of fibre and folate towards improvement in intakes emerged already in ‘scenario 70 g’. Overall, distribution shifts towards a higher probability of inadequate intakes of the studied nutrients were not observed.

**Conclusions:**

These results support the public health message to partly replace meat with legumes and may benefit nutrition policy actions towards sustainable diets in the Nordic countries and beyond.

According to the global reference diet for adults^(1)^ and dietary guidelines in many countries, including the Nordic countries^(2)^, there is a large consensus that diets rich in plant-based foods promote health and environmental sustainability, while the role of animal-based foods needs to be diminished. In terms of health, high red and processed meat consumption have been associated with an increased risk of several chronic diseases^(3)^, whereas there is longstanding evidence behind the protective role of plant foods^(4)^. In addition, findings on the advantages of legumes in ameliorating chronic diseases and their risk factors are accumulating^(5)^. Moreover, diets with less meat and more legumes have gained attention as a solution to decrease the environmental impact of food systems and diets^(6)^.

Meat is a good source of many nutrients and therefore diminishing its consumption calls for culturally accepted alternative ways to achieve a balanced and nutritionally adequate diet^(7,8)^. Globally, meat consumption is increasing and is currently exceeding the recommended levels in many countries^(9,10)^. In contrast, the consumption of legumes remains relatively low, especially in Western countries, implying a clear need to communicate their nutritional benefits as part of balanced diets^(11,12)^.

Legumes comprise pods and fruits of plants of the botanical families *Leguminosae* or *Favaceae*, the main subclasses being pulses (e.g. dry beans and peas, chickpeas, lentils), fresh legumes (green beans and peas) and oil-seed legumes (soybean, soy products, peanuts)^(5)^. Legumes, especially pulses, represent a good source of plant protein, fibre, vitamins and phytochemicals^(13,14)^. Several studies have shown that high consumption of pulses is associated with an overall better nutrient intake profile, suggesting that legume-rich diets are overall healthier^(15–17)^. However, nutrients derived mainly and easily from meat and other animal sources, such as vitamin B_12_ and bioavailable haem Fe, might pose a challenge in plant-rich diets^(18,19)^. The key question remains, how the replacement of red and processed meat with legumes impacts the nutrient intakes and nutrient adequacy of populations and population groups. To control for unintended outcomes of dietary transition on nutritional adequacy of the population, more research utilising substitution scenarios is needed.

Thus far only few studies have modelled the consequences of replacing red and processed meat with legumes on the nutrient intake and nutritional adequacy of diets in general adult populations. A recent study comprising about 2000 French adults showed that raising the quantity of pulses (including dry beans, dry peas, chickpeas and lentils) to the recommended level (57 g/d cooked, i.e. twice a week) in a replacement of an equivalent portion of meat improved several nutritional indicators^(20)^. For example, adherence to current French food-based dietary guidelines improved as assessed with a dietary score and the mean adequacy ratio which measures achievement of recommended intakes of 23 nutrients. In a Swedish study comprising about 1800 adults and modelling a 50 % reduction of meat and concomitant increase in pulse consumption (i.e. faba beans, yellow peas, gray peas, common beans and lentils) found that average intakes of energy, macronutrients and micronutrients remained within recommendations with improvements in average intakes of fibre and folate^(21)^. While these studies concentrated on average intakes or scores and ratios constructed thereof, less attention has been paid to the impact of replacement scenarios on the magnitude of population shares exposed to inadequate nutrient intakes. Moreover, studies thus far have mostly reported results in genders combined.

The understanding of the impact of legume-meat substitutions on the nutritional adequacy of diets needs to be reinforced to foster science-based public health messages on the nutritional benefits and risks of increasing legume consumption in place of meat. Moreover, this understanding is needed as the basis for nutrition policy directed to support the transition towards sustainable diets. The aim of the present study was to model the impact of partial replacement of red and processed meat with legumes on average intakes of selected nutrients. In addition, changes in population shares below dietary reference intakes of selected nutrients in Finnish women and men were explored.

## Methods

### Study population

We used the data of the National FinDiet 2017 Survey^(22,23)^ conducted as part of the FinHealth 2017 Study, which aimed to produce reliable data on health, well-being and functional capacity in Finnish adults^(24)^. The FinHealth 2017 Study comprised a nationally representative sample of 10 247 adults aged 18 years and over who received an invitation to a health examination alongside a background questionnaire. Altogether 30 % (*n* 3099) of the FinHealth sample belonged to the FinDiet 2017 subsample. Of these 59 % participated in the health examination and were eligible for the dietary data collection (i.e. two non-consecutive 24-hour dietary recalls). At the study clinic, 29 subjects refused or were not able to participate in the first dietary recall, 114 subjects were not reached for the second dietary recall and 16 subjects were excluded due to one or two incomplete dietary recalls. Eventually, 1655 adults aged 18–74 years (53 % of the invited) achieved full participation in the dietary data collection and formed the analytical data of the present study (875 women and 780 men).

### Socio-demographic and background variables

Subject’s sex and age were obtained from the sampling frame (Population Information system). The self-administered background questionnaire inquired on total years of education which was used to classify subjects into educational tertiles (low, middle, high) according to sex and birth year. This was done to adjust the classification for the extension of the basic education system and increase of average school years over the past decades.

### Diet

The dietary assessment method comprised two non-consecutive 24-hour dietary recalls from each subject carried out by uniformly trained dietary interviewers and a validated portion size picture book^(25,26)^ according to pan-European EU Menu methodology^(22,27)^. The first recall was conducted face-to-face during the health examination in January–May 2017 and the second recall via telephone during February–October 2017. The final data included all seasons and all days of the week, the weekdays covering 73 % and week-end days 27 %. The rate of energy underreporting ranged between 15 % and 23 % and energy overreporting was below 0·5 %^(22)^. Food consumption, energy and nutrient intakes were calculated using the in-house calculation software Finessi, which utilises the standard recipes, food composition information and food grouping of the Finnish national food composition database Fineli^®(28,29)^. In general, nutrient intakes were calculated from foods as consumed, i.e. nutrient losses during food preparation were considered for the following nutrients: vitamin A, beta carotene, vitamin C, thiamine, riboflavin, niacin, pyridoxine, folate and vitamin B_12_^(30)^. In this study, the average nutrient intakes based on the two recalls was used for the average nutrient intake analyses, and the two separate recalls when modelling usual intake distributions. Nutrient intakes from supplements were not considered. The selection of nutrients was based on the understanding of the current nutritional challenges of the Finnish adult population (e.g. fibre, saturated fat, folate)^(23)^ or nutrients that are readily available from red and processed meat (e.g. protein, fat, saturated fat, monounsaturated fat, vitamin A, thiamine, riboflavin, niacin, pyridoxine, vitamin B_12_, Cu, Fe, Se, Zn) or legumes (e.g. protein, fibre, folate, potassium, Mg, Cu, Fe).

### Red and processed meat

Red meat was defined as beef, pork, lamb, game, offal and processed meat as meat products including sausages and cold cuts. Consumption of red and processed meat was first calculated at the ingredient level by decomposing all meat-containing dishes according to standard recipes of the database^(31)^ and adding up with processed meats consumed as such. The ingredient consumption of red meat was converted into cooked red meat consumption by applying an average conversion factor of 0·7 (500 g cooked red meat corresponds to 700–750 g uncooked meat). For processed meat, no conversion factor was applied. Furthermore, to derive nutrient intakes from red and processed meat in the form consumed, nutrients derived from ingredient-level red meat were converted to nutrients derived from cooked red meat by applying average retention factors (i.e. retention factors averaged for different food preparation methods) for vitamin A, thiamine, riboflavin, niacin, pyridoxine, folate and vitamin B_12_^(30)^. For processed meat, no retention factors were applied.

### Legumes

Based on the most consumed legume foods, we designed an average recipe (i.e. legume food aggregate) that represented the consumption ratio of different types of legumes consumed by the Finnish adult population (Table 1). Cooked green peas represented the largest share (45 %) followed by green beans, kidney beans and soya mince (each with a 10 % share), as well as white beans, chickpeas, lentils and two popular legume-based meat alternatives (each with a 5 % share). This legume food aggregate and its nutrient content per 100 g (cooked) were used when carrying out the scenario analyses (see below). Overall, legume consumption reported in this article comprises all forms of fresh legumes (green peas or green beans), pulses (dry beans, chickpeas, lentils), soya products and legume-based plant-protein products. Moreover, legumes refer to both the legumes consumed as such and legumes from recipes.

**Table 1:** Most consumed legumes in the FinDiet 2017 Survey*, their relative contribution (%) to the legume aggregate used in the replacement scenarios and their nutritional composition per 100 g (cooked) based on the Finnish food composition database Fineli®†

	Green peas, boiled without salt‡	Soya mince, boiled without salt	Green bean, string bean, boiled‡	Kidney bean, boiled	Bean, white bean, boiled	Chickpea, boiled without salt	Red lentils, boiled	Härkis®, broad bean product§	Pulled oats||
Relative contribution (%)	45	10	10	10	5	5	5	5	5
Energy (kJ)	357	533	159	484	465	575	424	877	840
Protein (g)	6·4	18·8	2·2	8·6	8·3	8·4	7·6	17·0	30·0
Fat (g)	0·9	0·9	0·3	0·5	0·8	2·5	0·4	9·0	4·4
SFA (g)	0·19	0·10	0·07	0·07	0·08	0·30	0·00	0·59	0·38
MUFA (g)	0·10	0·16	0·01	0·04	0·04	0·60	0·00	5·01	2·32
Carbohydrate (g) ¶	10·8	8·0	5·1	15·3	14·0	17·6	15·6	13·1	8·1
Fibre (g)	3·5	5·7	2·6	7·4	7·0	5·0	1·9	4·0	3·7
Vitamin A (mg RE)	31·8	0·7	15·5	0·0	40·0	1·5	1·7	13·0	1·1
Thiamin (mg)	0·21	0·20	0·09	0·16	0·12	0·12	0·11	0·16	0·12
Riboflavin (mg)	0·04	0·08	0·05	0·06	0·05	0·06	0·04	0·11	0·03
Niacin (mg NE)	3·1	4·1	1·0	0·6	2·4	1·9	1·6	2·2	1·0
Pyridoxine (mg)	0·18	0·26	0·11	0·119	0·18	0·14	0·11	0·1	0·04
Folate (mg)	46·9	80·8	59·6	129·0	117·3	172·0	5·0	51·6	8·8
K (mg)	420	949	340	401	365	290	220	283	390
Mg (mg)	45	117	23	45	49	48	26	50	35
Cu (mg)	0·3	0·8	0·1	0·2	0·2	0·3	0·2	0·2	0·1
Fe (mg)	2·3	5·2	0·7	2·9	1·9	2·9	2·4	1·9	6·0
Se (mg)	0·7	1·3	0·3	1·2	0·4	5·0	2·0	1·9	2·0
Zn (mg)	1·5	1·9	0·4	1·1	0·8	1·5	1·0	0·8	0·8

* Valsta *et al.* 2018^(23)^.

† Finnish Institute for Health and Welfare 2022^(28)^.

‡ Varieties harvested as green.

§ Härkis® contains faba beans and pea protein.

|| Pulled oats contain oat, pea protein and faba bean protein.

¶ Available carbohydrate excluding fibre.

### Statistical methods

We designed three theoretical replacement scenarios anchored in cut-off values of red and processed meat: (1) ‘scenario 70 g’ in which all subjects attained a consumption of red and processed meat of no more than 70 g/d corresponding to the Finnish dietary guideline (max. 500 g/week)^(32)^, (2) ‘scenario 50 g’ in which all subjects attained a consumption of red and processed meat of no more than 50 g/d corresponding to the recent Danish food based dietary guidelines (max. 350 g/week)^(33)^ and (3) ‘scenario 30 g’ in which all subjects attained a consumption of red and processed meat of no more than 30 g/d which corresponds to the upper limit of the recommended consumption range of the EAT Lancet recommendation (max. 200 g/week)^(1)^. In the scenarios, for all subjects with consumption values above the scenario cut-offs, their red and processed meat quantity (in grams) exceeding the cut-off was replaced with the legume food aggregate (see above). The consumption of other food groups remained unchanged across scenarios.

We computed means and their 95 % CI in the reference (FinDiet 2017) and in each of the three scenarios. The SPADE method^(34)^ was used to estimate usual intake distributions of selected nutrients and to assess the population shares below dietary reference values given in the Nordic and Finnish nutrition recommendations^(2,32)^. When an average requirement (AR) value was not available for a given nutrient,^(2)^ the recommended intake (RI)^(32)^ was used. The 95 % CI for the population shares below dietary reference values were computed using the bootstrap method with 500 iterations. The differences between the reference and the scenarios (both when comparing means and when comparing the population shares below dietary reference values) could not be tested by standard statistical tests as the data have been altered by study design. Therefore, meaningful differences were evaluated by non-overlapping 95 % CI^(35,36)^. All analyses were conducted separately in women and men, since in Finland high red and processed meat consumption is more common in men compared to women. Moreover, women tend to have an overall healthier diet compared to men^(23)^. Survey weights were used to adjust for non-participation bias and to improve the generalisability of the results to the Finnish adult population^(37)^.

## Results

In the FinDiet 2017 Survey (reference), the average consumption of legumes was 13 g/d in women and 12 g/d in men (Table 2). Younger age groups (adults in the age range 18−44 years) and those belonging to the highest educational group tended to consume more legumes than the others. Men consumed twice as much red and processed meat (114 g/d) on average as women (58 g/d). Subjects in the highest educational group tended to consume less red and processed meat compared to the lower educated groups.

**Table 2:** Daily consumption of legumes as well as red and processed meat in the FinDiet 2017 Survey* by sex, age and education (Means and 95 % CI for means)

	Legumes (g/d)†		Red and processed meat (g/d) ‡	
	Mean	95 % CI for mean	Mean	95 % CI for mean
Women				
All (*n* 875)	13	10, 16	58	55, 62
Age group				
18–24 (*n* 52)	23	9, 38	56	39, 73
25–44 (*n* 259)	15	11, 19	61	55, 67
45–64 (*n* 317)	10	7, 12	59	54, 64
65–74 (*n* 247)	9	6, 12	54	49, 60
Education				
Low (*n* 269)	11	8, 15	66	60, 72
Middle (*n* 305)	14	8, 19	58	52, 65
High (*n* 285)	15	11, 19	50	45, 56
Men				
All (*n* 780)	12	9, 15	114	108, 121
Age group				
18–24 (*n* 47)	20	5, 36	137	115, 159
25–44 (*n* 221)	15	10, 21	118	107, 130
45–64 (*n* 308)	9	6, 11	111	102, 121
65–74 (*n* 204)	7	5, 9	96	85, 108
Education				
Low (*n* 259)	7	5, 10	122	110, 134
Middle (258)	14	7, 20	122	111, 133
High (*n* 256)	15	10, 21	96	84, 109

* Valsta *et al.* 2018^(23)^.

† Legumes comprises all forms fresh legumes (green peas or green beans), pulses (dry beans, chickpeas, lentils), soya products and legume-based plant-protein products.

‡ Red meat comprises beef, pork, lamb, game and offal and processed meat comprises sausages and cold cuts.

### Replaced quantities of red and processed meat

**Fig. 1:**
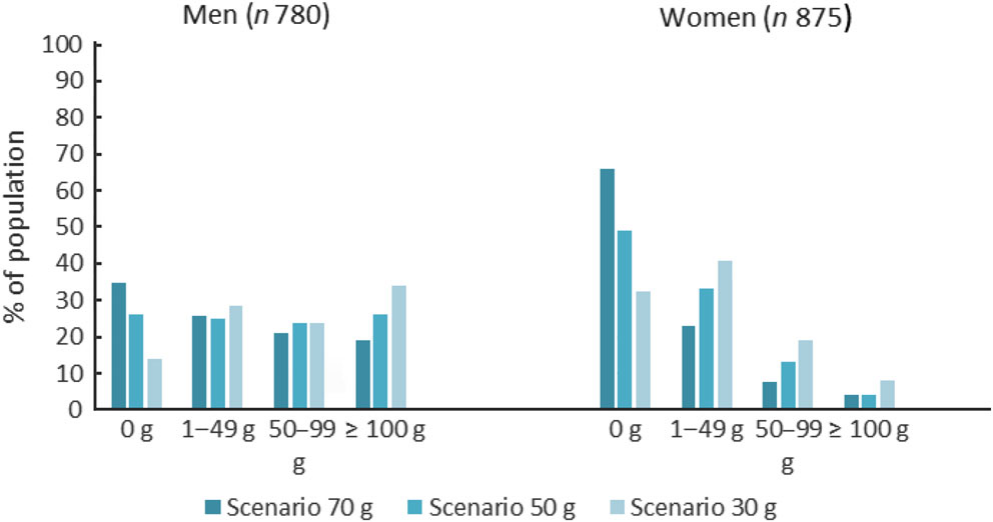
Proportion of population groups to decrease red and processed meat intake at different quantities (g) by sex and replacement scenario based on the FinDiet 2017 Survey^(23)^

The proportion of men subject to the reduction of red and processed meat ranged from 67 % (‘scenario 70 g’) to 87 % (‘scenario 30 g’), the corresponding values for women ranged from 34 % to 67 % (data not shown). Across scenarios, the average daily quantities of red and processed meat to be replaced by legumes ranged between 83 g and 100 g in men and 42 g and 52 g in women. In ‘scenario 70 g’, 19 % of the men, and in ‘scenario 30 g’, 34 % of the men were subject to replacing a daily quantity of 100 g or more of red and processed meat, while this was the case for 4 % and 8 % of the women, respectively (Fig. 1).

### Consumption of legumes and red and processed meat

When shifting from the reference to ‘scenario 70 g’, women’s legume consumption doubled from 13 g/d to 27 g/d and quadrupled to 48 g/d when shifting from the reference to ‘scenario 30 g’. In men, legume consumption was almost sixfold in ‘scenario 70 g’ (68 g/d) and eightfold in ‘scenario 30 g’ (98 g/d), compared to the reference (12 g/d) (Table 3). When shifting from the reference to ‘scenario 70 g’, the average consumption of red and processed meat decreased in women from 58 g/d to 44 g/d (25 % decrease) and further to 24 g/d in ‘scenario 30 g’ (60 % decrease compared to the reference). In men, red and processed meat consumption decreased from 114 g/d (reference) to 58 g/d in ‘scenario 70 g’ (50 % decrease) and further to 28 g/d in ‘scenario 30 g’ (75 % decrease compared to the reference).

**Table 3:** Mean daily consumption of legumes† and red and processed meat‡ as well as daily intakes of energy and macronutrients in the reference scenario (FinDiet 2017§) and replacement scenarios by sex (Means with their 95 % CI)

	Reference scenario		Scenario 70 g		Scenario 50 g		Scenario 30 g	
	Mean	95 % CI	Mean	95 % CI	Mean	95 % CI	Mean	95 % CI
Women (*n* 875)								
Legumes (g)	13	11, 16	27*	25, 30	36*	33, 39	48*	45, 51
Red and processed meat (g)	58	55, 62	44*	42, 46	36*	34, 37	24*	23, 25
Energy (MJ)	7·4	7·2, 7·5	7·3	7·1, 7·4	7·2	7·1, 7·4	7·2	7·0, 7·3
Protein (E%)	17·5	17·2, 17·7	17·3	17·0, 17·6	17·2	16·9, 17·5	17·0	16·7, 17·3
Fat (E%)	37·8	37·2, 38·4	37·2	36·6, 37·8	36·8	36·2, 37·4	36·3*	35·6, 36·9
Saturated fat (E%)	14·4	14·0, 14·7	14·1	13·7, 14·4	13·9	13·6, 14·2	13·6*	13·3, 14·0
Monounsaturated fat (E%)	14·3	14·0, 14·6	14·0	13·7, 14·3	13·8	13·5, 14·1	13·5*	13·2, 13·8
Carbohydrate (E%)||	42·4	41·8, 43·1	43·1	42·5, 43·8	43·6	42·9, 44·2	44·2*	43·6, 44·9
Fibre (g/MJ)	2·9	2·8, 2·9	3·0	2·9, 3·0	3·0*	3·0, 3·1	3·1*	3·1, 3·2
Men (*n* 780)								
Legumes (g)	12	9, 15	68*	61, 75	82*	75, 90	98*	91, 106
Red and processed meat (g)	114	108, 121	58*	57, 60	44*	43, 45	28*	27, 28
Energy (MJ)	9·4	9·1, 9·7	9·1	8·9, 9·4	9·1	8·8, 9·3	9·0	8·7, 9·2
Protein (E%)	18·0	17·7, 18·3	17·5	17·2, 17·9	17·4*	17·1, 17·7	17·2*	16·9, 17·5
Fat (E%)	38·7	38·1, 39·2	36·8*	36·3, 37·3	36·2*	35·7, 36·7	35·6*	35·0, 36·1
Saturated fat (E%)	15·1	14·8, 15·4	14·2*	13·9, 14·5	13·9*	13·6, 14·2	13·6*	13·3, 13·9
Monounsaturated fat (E%)	14·6	14·3, 14·8	13·6*	13·4, 13·8	13·3*	13·1, 13·6	13·0*	12·7, 13·2
Carbohydrate (E%)||	41·3	40·7, 42·0	43·4*	42·9, 44·0	44·1*	43·5, 44·6	44·8*	44·3, 45·4
Fibre (g/MJ)	2·5	2·4, 2·6	2·8*	2·7, 2·9	2·9*	2·8, 3·0	3·0*	2·9, 3·1

* Meaningfully different from the reference scenario based on non-overlapping 95 % CI around the mean (Quann *et al.* 2015^(35)^, Cifelli *et al.* 2016^(36)^).

† Legumes comprises all forms of fresh legumes (green peas or green beans), pulses (dry beans, chickpeas, lentils), soya products and legume-based plant-protein products.

‡ Red meat comprises beef, pork, lamb, game and offal and processed meat comprises sausages and cold cuts.

§ Valsta *et al.* 2018^(23)^.

|| Available carbohydrate excluding fibre.

### Average intakes of energy and nutrients

Despite a decreasing energy intake pattern across scenarios, no meaningful changes in energy intakes compared to the reference were observed. Overall, results for absolute and energy-adjusted (E% or g/MJ) intakes of macronutrients, vitamins and minerals in each of the scenarios were mostly similar, and therefore only energy-adjusted results are shown (Tables 3 and 4).

**Table 4:** Mean daily intakes of vitamins and minerals in the reference scenario (FinDiet 2017†) and replacement scenarios by sex (Means with their 95 % CI)

	Reference scenario		Scenario 70 g		Scenario 50 g		Scenario 30 g	
	Mean	95 % CI for mean	Mean	95 % CI for mean	Mean	95 % CI for mean	Mean	95 % CI for mean
Women (*n* 875)								
Vitamin A (ug RAE/MJ)	105·1	98·8, 111·4	104·7	98·9, 110·4	103·7	98·5, 108·8	102·5	98·1, 107·0
Thiamin (mg/MJ)	0·15	0·14, 0·15	0·15	0·14, 0·15	0·15	0·14, 0·15	0·15	0·14, 0·15
Riboflavin (mg/MJ)	0·22	0·22, 0·23	0·22	0·22, 0·23	0·22	0·22, 0·23	0·22	0·22, 0·23
Niacin equivalent (mg/MJ)	4·0	4·0, 4·1	4·0	3·9, 4·0	3·9	3·9, 4·0	3·9*	3·8, 3·9
Pyridoxine (mg/MJ)	0·25	0·25, 0·26	0·25	0·24, 0·26	0·25	0·24, 0·26	0·25	0·24, 0·26
Folate (ug/MJ)	30·9	30·0, 31·8	32·2	31·4, 33,1	33·1*	32·2, 33·9	34·3*	33·5, 35·1
Vitamin B_12_ (ug/MJ)	0·69	0·66, 0·72	0·67	0·64, 0·70	0·66	0·63, 0·69	0·64	0·61, 0·66
K (mg/MJ)	0·48	0·47, 0·49	0·49	0·48, 0·50	0·50*	0·50, 0·51	0·51*	0·50, 0·52
Mg (mg/MJ)	47·2	46·5, 48·0	48·2	47·4, 48·9	48·8*	48·1, 49·5	49·7*	48·9, 50·5
Cu (mg/MJ)	0·159	0·155, 0·163	0·164	0·160, 0·167	0·167*	0·163, 0·170	0·170*	0·167, 0·174
Fe (mg/MJ)	1·36	1·33, 1·40	1·39	1·36, 1·42	1·41	1·41, 1·44	1·43*	1·40, 1·46
Se (ug/MJ)	9·5	9·3, 9·8	9·3	9·1, 9·6	9·2	8·9, 9·5	9·0*	8·7, 9·2
Zn (mg/MJ)	1·34	1·32, 1·36	1·32	1·30, 1·34	1·31	1·29, 1·33	1·28*	1·27, 1·30
Men (*n* 780)								
Vitamin A (ug RAE/MJ)	100·7	92·1, 109·4	99·4	92·6, 106·3	98·2	92·3, 104·1	96·7	91·8, 101·6
Thiamin (mg/MJ)	0·15	0·14, 0·15	0·15	0·14, 0·15	0·15	0·14, 0·15	0·15	0·14, 0·15
Riboflavin (mg/MJ)	0·21	0·21, 0·22	0·21	0·21, 0·22	0·21	0·21, 0·22	0·21	0·21, 0·22
Niacin equivalent (mg/MJ)	4·2	4·1, 4·3	4·0*	4·0, 4·1	4·0*	3·9, 4·1	3·9*	3·8, 4·0
Pyridoxine (mg/MJ)	0·2	0·2, 0·3	0·2	0·2, 0·3	0·2	0·2, 0·3	0·2	0·2, 0·3
Folate (ug/MJ)	26·9	26·2, 27·7	31·2*	30·4, 31·9	32·4*	31·6, 33·1	33·8*	33·0, 34·5
Vitamin B_12_ (ug/MJ)	0·72	0·68, 0·76	0·67	0·63, 0·71	0·65	0·61, 0·68	0·63*	0·59, 0·66
K (mg/MJ)	0·44	0·43, 0·45	0·48*	0·47, 0·49	0·49*	0·48, 0·50	0·51*	0·50, 0·52
Mg (mg/MJ)	44·9	44·0, 45·7	47·9*	47·0, 48·7	48·7*	47·9, 49·6	49·8*	48·9, 50·7
Cu (mg/MJ)	0·14	0·14, 0·15	0·16*	0·15, 0·16	0·16*	0·16, 0·17	0·17*	0·16, 0·17
Fe (mg/MJ)	1·26	1·23, 1·30	1·35*	1·32, 1·39	1·38*	1·35, 1·41	1·41*	1·38, 1·44
Se (ug/MJ)	9·6	9·3, 9·8	8·9*	8·7, 9·2	8·7*	8·5, 9·0	8·5*	8·2, 8·7
Zn (mg/MJ)	1·39	1·37, 1·42	1·34*	1·31, 1·36	1·32*	1·29, 1·34	1·29*	1·27, 1·32

* Meaningfully different from the reference scenario based on non-overlapping 95 % CI around the mean (Quann *et al.* 2015^(35)^, Cifelli *et al.* 2016^(36)^).

† Valsta *et al.* 2018^(23)^.

Partial replacement of red and processed meat with legumes resulted in decreased intakes of total fat (E%), saturated fat (E%) and monounsaturated fat (E%) and concomitant increase in intakes of carbohydrate (E%) and fibre (g/MJ) (Table 3). In men, these changes were observed already in ‘scenario 70 g’, whereas in women not until ‘scenario 30 g’ (exception fibre (g/MJ) increased already in ‘scenario 50 g’). A decrease in protein intake (E%) was evident only in men (‘scenario 50 g’ and ‘scenario 30 g’). Regarding vitamins and minerals, increases in folate (μg/MJ), K (mg/MJ), Mg (mg/MJ), Cu (mg/MJ) and Fe (mg/MJ) were observed for both sexes in ‘scenario 30 g’ with concomitant decreases of niacin (mg/MJ), Se (μg/MJ) and Zn (mg/MJ) (Table 4). In men, most of these results were evident already in ‘scenario 70 g’ and in women in ‘scenario 50 g’. For vitamin B_12_, a decrease was observed only in men (‘30 g scenario’). For vitamin A, thiamine, riboflavin and pyridoxine, no meaningful changes in average intakes across scenarios were observed.

When analysing absolute intakes of nutrients, the scenario results resembled those obtained with the energy-adjusted nutrients. The only exceptions were that in women for ‘scenario 50 g’ meaningful increases in intakes of fibre (g/d) and folate (μg/d) were not evident, and for ‘scenario 30 g’, a decrease in protein (g/d) intake was observed (data not shown). In men, for ‘scenario 70 g’, meaningful increases in intakes of Mg (mg/d) and Cu (mg/d) were not seen, and the decrease in vitamin B_12_ intake (μg/d) was evident already in ‘scenario 50 g’. The decrease in thiamine intake (mg/d) was seen only in men and only for the absolute intake value in ‘scenario 30 g’ (data not shown).

### Evaluation of intakes against dietary reference values

**Fig. 2:**
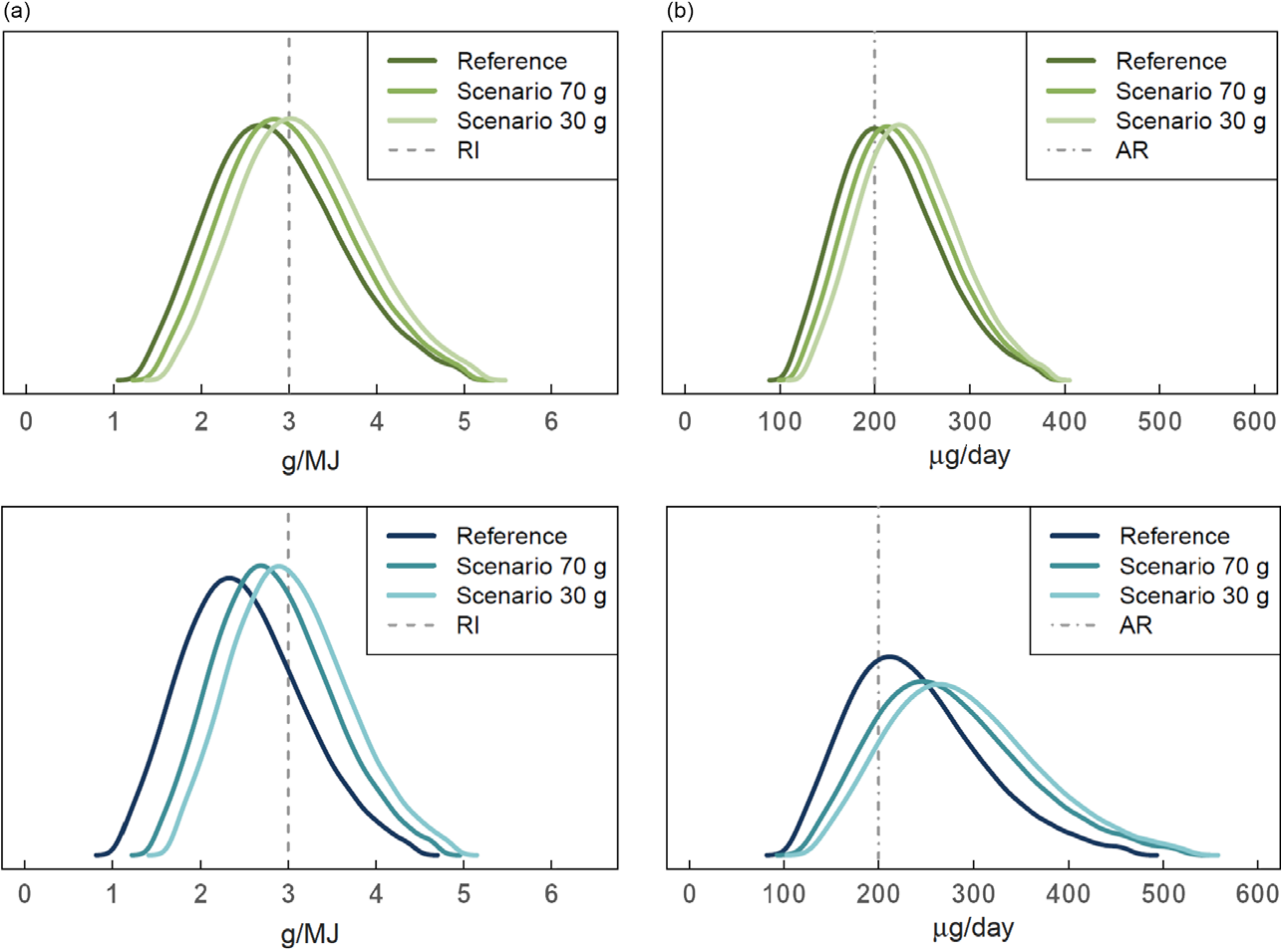
Usual intake distributions for fibre (a) and folate (b) in Finnish women (upper panel) and men (lower panel) in the national FinDiet 2017 Survey (reference) and two scenarios in which all individuals limit their consumption of red and processed meat to no more than 70 g/d (scenario 70 g ; corresponding to the Finnish dietary guideline of max. 500 g/week)^(32)^ or to no more than 30 g/d (scenario 30 g; corresponding to the Planetary Health Diet recommendation of max. 200 g/week)^(1)^. In the scenarios, for each individual the amount exceeding the limit was replaced by the same amount of legumes. RI , recommended intake, AR , average requirement. Dietary reference values are based on the Nordic nutrition recommendations^(2)^ and the Finnish nutrition recommendations^(32)^

When evaluating population shares below the RI or the AR for selected nutrients in the extreme scenarios (‘scenario 70 g’ and ‘scenario 30 g’) compared to the reference meaningful shifts towards improved intakes of fibre and folate were observed already in ‘scenario 70 g’ (Fig. 2, Table 5). In women, the population share below the RI for fibre (3 g/MJ/d) was 7 percentage points smaller in ‘scenario 70 g’ and 16 percentage points smaller in ‘scenario 30 g’ compared to the reference (Table 5). The corresponding percentage points in men were 15 and 26. Regarding the population shares with folate intakes below the AR (200 μg/d), the decreases ranged between 9 and 16 percentage points in women and between 15 and 19 percentage points in men for ‘scenario 70 g’ and ‘scenario 30 g’, respectively. In men, the population share with saturated fat intakes below the recommended daily maximum (10 E%) was 7 percentage points higher in ‘scenario 30 g’ compared to the reference (FinDiet 2017). In addition, decreases in the population shares below the RI for potassium were observed for women (9 percentage points in ‘scenario 30 g’) and men (8 percentage points in ‘scenario 70 g’ and 12 percentage points in ‘scenario 30 g’). For the rest of the studied minerals, there were no meaningful changes in population shares below the AR in the scenarios compared to the reference.

**Table 5:** Evaluation of population shares below dietary reference intakes† based on usual intake estimation in the reference scenario (FinDiet 2017‡) and two replacement scenarios by sex

		Reference scenario		Scenario 70 g		Scenario 30 g	
	Recommendation	Estimate	95 % CI	Estimate	95 % CI	Estimate	95 % CI
		% < RI		% < RI		% < RI	
Saturated fat (E%)							
Women	max. 10 (RI)	6	3, 8	7	5, 10	10	6, 13
Men	max. 10 (RI)	3	2, 5	6	4, 9	10*	7, 13
Fibre (g/MJ)							
Women	3 (RI)	59	56, 63	52*	48, 56	43*	39, 47
Men	3 (RI)	76	73, 80	61*	57, 65	50*	45, 53
K (mg)							
Women	3·1 (RI)	35	31, 39	30	27, 34	26*	23, 30
Men	3·5 (RI)	34	31, 37	26*	23, 30	22*	19, 26
		% < AR		% < AR		% < AR	
Folate (ug)							
Women	200 (AR)	40	36, 44	31*	27, 35	24*	19, 28
Men	200 (AR)	31	26, 34	16*	13, 20	12*	8, 14
Niacin equivalent (mg)							
Women	12 (AR)	0·02	0·00, 0·09	0·02	0·00, 0·10	0·04	0·01, 0·16
Men	15 (AR)	0·07	0·02, 0·17	0·07	0·02, 0·15	0·14	0·04, 0·29
Vitamin B_12_ (ug)							
Women	1·4 (AR)	0·04	0·00, 0·24	0·06	0·00, 0·31	0·12	0·01, 0·53
Men	1·4 (AR)	0·02	0·00, 0·06	0·05	0·01, 0·18	0·17	0·02, 0·45
Fe (mg)							
Women	10 (AR)§	57	54, 61	55	51, 59	53	49, 56
Men	7 (AR)	5	3, 7	4	2, 5	3	2, 4
Se (ug)							
Women	30 (AR)	0·2	0·1, 0·6	0·3	0·1, 0·8	0·7	0·3, 1·3
Men	35 (AR)	0·5	0·2, 0·9	0·7	0·3, 1·3	1·6	0·7, 2·7
Zn (mg)							
Women	5 (AR)	0·4	0·1, 1·0	0·6	0·2, 1·2	1·0	0·5, 1·7
Men	6 (AR)	0·9	0·4, 1·5	1·2	0·6, 2·0	1·9	1·1, 2·8

RI , recommended intake, AR , average requirement.

* Population shares are meaningfully different compared to reference (based on the non-overlapping 95 % CI around the point estimate as estimated using 500 bootstrap samples).

† Dietary reference intakes are based on the Nordic nutrition recommendations^(2)^ and the Finnish nutrition recommendations^(32)^.

‡ Valsta *et al.* 2018^(23)^.

§ Not suitable for the evaluation of intake adequacy for women in reproductive age. For postmenopausal women, the AR is 6 mg/d.

## Discussion

The aim of this study was to model the theoretical impact of partial replacement of red and processed meat with legumes on the nutrient intakes and on the population shares below dietary reference intakes in the Finnish adult population. The replacement proved beneficial in terms of saturated fat and fibre intakes, as well as intakes of folate, and several vitamins and minerals for which legumes are a good source of. Based on the evaluation of population shares below dietary reference intakes, the replacement scenarios introduced meaningful shifts of the nutrient intake distributions towards the improvement of population-level intakes of fibre and folate. Meaningful shifts to the opposite direction were not observed, implying that across scenarios the probability of inadequacy remained stable for the studied nutrients.

The Nordic food culture carries a great potential to substantially increase legume consumption, as the role of legumes in current diets is considered small^(12,21)^. Furthermore, the need to decrease meat consumption is evident from the public health perspective in the Nordic countries and Europe^(10)^. Livestock and dairy production are embedded in the Nordic food systems^(38)^, which influences the diet of people in these regions. Meat and milk products, in addition to cereals and vegetables, are important sources of many nutrients. This was also highlighted in our results: On the population level, partial replacement of red and processed meat with legumes, even for meat consumption levels of the EAT Lancet Planetary health diet, did not introduce a risk to inadequate intakes of the selected nutrients (e.g. niacin, vitamin B_12_, Fe, Se, Zn) given that milk products and cereals, vegetables, poultry and fish continue to be part of the diet.

Overall, the results of this study are in accordance with earlier scenario studies from Sweden^(21)^ and France^(20)^ which also focussed on the replacement of meat with legumes. Röös *et al.* ^(21)^ found a 50 % reduction of meat and corresponding increase in pulses to be beneficial regarding fibre and folate intakes. Gazan *et al.* ^(20)^ showed that the increase in the consumption of pulses (twice a week) in place of meat improved several nutritional indicators. However, our study provides an additional dimension by showing that the population shares below RI or AR diminished substantially for fibre and folate already in ‘scenario 70 g’ both in women and men. This suggests that the sole reduction of red and processed meat to the level of 500 g/week and the replacement of the extra red and processed meat with legumes may introduce improvements in intakes of fibre and folate – both critical in the current Finnish diet^(23)^. Therefore, our results provide an encouraging public health message in terms of step-by-step tackling of current nutritional challenges in Nordic adult populations and at the same time support the shift towards the environmental sustainability targets.

In general, despite our focus on legumes as the meat substitute, our results are in line with earlier studies utilising plant-based substitutes more broadly. In a study comprising 2102 Dutch adults, 30 % or 100 % replacements of meat and dairy products by plant-based alternatives proved beneficial for intakes of SFA and fibre whereas the 100 % replacement introduced increasing inadequate intakes of e.g. vitamin A, thiamine, vitamin B_12_ and Z^(39)^. Therefore, the inclusion of milk products along with meat could have reinforced the outcomes of our substitution analysis. Furthermore, by using a scoring system for probability of adequate intakes in the French national food consumption survey (*n* 2121), plant-based substitutes that included legumes appeared more nutritionally adequate compared to cereal-based substitutes^(40)^. However, regardless of the type of the plant-based substitute they reported, similarly to us, improvements of adequacy for fibre, folate and saturated fat, but also lower adequacies for vitamin B_12_ as well as bioavailable Fe and Zn were found. In contrast, a scenario analysis using NHANES 2007–2010 data (*n* 17 387) showed that doubling the consumption of protein-rich plant based-foods (i.e. beans, peas, legumes, nuts, seeds and soy products) and equal decrease of animal products did not impact the nutrient intakes due to low consumption of protein-rich plant foods in the baseline diet^(36)^. However, a doubling of currently consumed plant-based foods decreased the share of the American adult population below estimated AR for e.g. folate, Mg and Fe suggesting that increasing plant-based foods overall is of importance.

Taken together, scenario analyses provide valuable insight in the benefits and risks of shifting to more plant-based diets but include differing built-in assumptions and analytical choices that hamper their direct comparability. In the case of plant-based meat alternatives, the types of alternative foods are of importance. The continuously increasing assortment of plant-based meat substitutes creates room for further investigation as there are both healthy and unhealthy options on the market. In this regard, studies based on ingredient-level foods (i.e. dishes decomposed to their ingredients) as the substitutes, like in the present study, provide a useful additional viewpoint. Furthermore, studies conducted thus far highlight the central role of the background diet for the results obtained. Since diets are, by far, food culture dependent, efficient monitoring of food consumption and nutrient intakes of populations in the era of shifts towards more sustainable diets remain a necessity.

Since the food-based dietary guidelines for legumes vary considerably between European countries^(41)^ and often lack a target amount in grams, we chose to build our scenarios on the red and processed meat guidelines^(1,32,33)^. Across our scenarios, it was evident that men were subject to a greater demand to decrease their consumption to achieve recommended consumption levels followed by more pronounced outcomes of the scenario analyses. As expected, the consumption of legumes increased considerably closer to the EAT Lancet reference of 75 g/d when standardised to a 2500 kcal diet, which should be an achievable target. Moreover, a recent systematic review of randomised controlled trials suggested that metabolic and health benefits of pulses may be achieved with daily consumption ranging between 54–360 g/d^(42)^. The practical feasibility of achieving high levels of legume consumption requires additional focus on using legumes in cooking practices both in homes and in public catering services. Furthermore, an established role of industrial legume-based meat substitutes in diets is key. These are globally recognised means to support the shift to legume-rich diets^(6,43)^.

A clear need to increase legume consumption has been recognized^(11,21)^. At the same time, the important role meat and meat products, not only in terms of protein intake, but also intake of other nutrients has been highlighted^(7)^. When meat consumption is radically reduced, higher probability of inadequate intakes may not necessarily be observed in the general population but may pose specific population groups at higher risk of nutritional inadequate diets. In Finland, the subjective importance of meat has been found to be a determinant of high red and processed meat consumption – the phenomenon being pronounced in men, those living in rural areas, and in subjects with lower education^(44,45)^. Similar findings have emerged from other countries^(46)^ and suggest the need to focus intervention on these population groups to reduce meat and increase legume consumption. Fortunately, there is recent data to show that increasing pulse consumption as plant-based meat alternative is a promising direction also from the viewpoint of societal acceptance^(47)^. Based on our results, it seems that young adults and subjects with higher education are leading the way to legume-rich diets. Overall, clearly formulated food-based dietary guidelines and their effective implementation are a requisite for increasing legume consumption^(43)^.

### Strengths and limitations

The national FinDiet 2017 Survey data, gathered according to EU Menu guidance, is one of the strengths of this analysis. Nationally representative sampling, a relatively high participation rate as well as analysis utilising survey weights improve the generalisability of our findings to the Finnish adult population and Nordic populations as appropriate. In addition, the harmonised EU Menu methodology eases the comparison to other European studies.

We used 24-hour recall data from two non-consecutive days, which is well-suited for reporting average consumption within a population. In the evaluation of population shares against dietary reference values, we were able to apply usual intake modelling using the SPADE program where intakes from at least 2 days are required^(36)^. In general, usual intake modelling removes the intra-individual variation apparent in short-term measurements, thus providing a more realistic distribution of the average long-term intake and improving the evaluation of population shares against dietary reference values. The earlier studies on partial replacement of red and processed meat with legumes^(20,21)^ used national dietary surveys as their reference diet, like our study, but did not consider the population shares below/above dietary reference values – which is key in understanding the distribution of nutrient intakes in the population.

Legume types currently consumed in Finland were the basis of our replacement strategy. This is considered a strength since these legume types are already culturally accepted. Most of the earlier studies showing nutritional benefits of increased legume consumption or replacement of meat with legumes concentrated on pulses as the legume subtype^(17,20,21)^. Giving more emphasis on pulses might have resulted in even greater nutritional benefits in our modelling study. However, it should be noted that legume consumption is overall low in Finland, like other Nordic countries^(12,21)^. Thus, the increase in consumption of any type of legumes should be encouraged, and more specific dietary guidance around legumes should be put purposefully in place. Currently, there are no specified recommendations for different legume types in the Finnish food-based dietary guidelines^(32)^.

As to weaknesses, critical nutritional issues related to an increased consumption of legumes, which our study did not consider, include the high concentration of antinutrients (e.g. lectin) in legumes which place the processing and cooking methods into the spotlight^(48)^. In addition, legumes contain a wealth of bioactive compounds that may hamper the bioavailability of certain nutrients, including Fe and Zn^(19)^. Earlier studies have shown that substituting plant-based foods or legumes for meat introduce lower adequacy or lower average intake of bioavailable Fe and Zn^(20,40)^. Like our result for men, Seves *et al.* showed an increase in total Fe intake across their replacement scenarios suggesting an impact of non-haem Fe found in plant foods^(39)^. Similar findings were obtained in a recent Finnish 12-week randomised controlled trial focussing on the replacement of animal-based protein with plant-based protein^(18)^. Future studies should take the aspect of bioavailability increasingly into consideration, especially in studies concerning population groups vulnerable to nutritional deficiencies of these minerals including premenopausal women, strict vegetarians/vegans, young children and the elderly. Overall, nutritional consequences of large dietary shifts should be studied, not only in the general adult population, but also throughout the life cycle.

The present study was a theoretical approach focussing solely on the partial replacement of red and processed meat with legumes without considering changes in the consumption of other animal-based foods. This was for instance the reason behind the modest or non-existent impacts of our replacement scenarios on the intakes of vitamin B_12_, niacin, pyridoxine, thiamine and riboflavin. According to the FinDiet 2017 Survey, when considering intake from food only, 25 % of women and 36 % of men are below the AR for thiamine, and 9 % of women and 18 % of men are below AR for riboflavin^(23)^. Thus, the intakes of these vitamins should be monitored more closely as the anticipated more comprehensive shift to plant-based diets proceeds. Individuals following strict vegetarian diets are at risk for developing vitamin B_12_ deficiency. Therefore, food fortification and supplementation regimens as well as new technologies in the production of plant foods may prove critical in supporting balanced nutrition of some population groups in future^(49)^.

## Conclusions

Our modelling results suggest that partial replacement of red and processed meat with legumes has great potential to improve intakes of critical nutrients on the population level without increasing the probability of inadequate intakes of nutrients that are well derived from red and processed meat. The replacement strategy represents one feasible step on the way to sustainable diets. This can be regarded as encouraging from both health and environment perspectives. While giving room for studies with more complex replacement scenarios, our results may prove useful in both national and Nordic food system transformation planning and implementation.
